# Optimisation of *ex vivo *memory B cell expansion/differentiation for interrogation of rare peripheral memory B cell subset responses

**DOI:** 10.12688/wellcomeopenres.11386.2

**Published:** 2018-01-24

**Authors:** Luke Muir, Paul F. McKay, Velislava N. Petrova, Oleksiy V. Klymenko, Sven Kratochvil, Christopher L. Pinder, Paul Kellam, Robin J. Shattock

**Affiliations:** 1 Department of Mucosal Infection and Immunity, Imperial College London, London, W2 1PG, UK; 2 The Wellcome Trust Sanger Institute, Cambridge, CB10 1SA, UK; 3 Department of Chemical and Process Engineering, University of Surrey, Guildford, GU2 7XH, UK; 4 Kymab Ltd., Cambridge, CB22 3AT, UK

**Keywords:** Memory B cell subsets, Human, Vaccination, Ex vivo expansion/differentiation, Design of Experiments

## Abstract

***Background**:* Human memory B cells play a vital role in the long-term protection of the host from pathogenic re-challenge. In recent years the importance of a number of different memory B cell subsets that can be formed in response to vaccination or infection has started to become clear. To study memory B cell responses, cells can be cultured *ex vivo,* allowing for an increase in cell number and activation of these quiescent cells, providing sufficient quantities of each memory subset to enable full investigation of functionality. However, despite numerous papers being published demonstrating bulk memory B cell culture, we could find no literature on optimised conditions for the study of memory B cell subsets, such as IgM ^+^ memory B cells.

***Methods**:* Following a literature review, we carried out a large screen of memory B cell expansion conditions to identify the combination that induced the highest levels of memory B cell expansion. We subsequently used a novel Design of Experiments approach to finely tune the optimal memory B cell expansion and differentiation conditions for human memory B cell subsets. Finally, we characterised the resultant memory B cell subpopulations by IgH sequencing and flow cytometry.

***Results**: *The application of specific optimised conditions induce multiple rounds of memory B cell proliferation equally across Ig isotypes, differentiation of memory B cells to antibody secreting cells, and importantly do not alter the Ig genotype of the stimulated cells.

***Conclusions**:* Overall, our data identify a memory B cell culture system that offers a robust platform for investigating the functionality of rare memory B cell subsets to infection and/or vaccination.

## Introduction

The B cell response plays a vital role in the defence against a variety of pathogens encountered throughout life. B cell responses are commonly categorised into two distinct subgroups known as “T cell-dependent” and “T cell-independent” responses. In T cell-dependent B cell responses, B cells are typically activated through recognition of their cognate antigen combined with cytokine and CD40 stimulation in the form of “T cell help” in the secondary or tertiary lymphoid tissues. Upon activation there are a number of differentiation pathways available to these B cells, with the three major options being: 1) to become short lived plasma cells, capable of secreting antibody in response to initial infection; or undergo clonal expansion, somatic hypermutation (SHM) and class switch recombination (CSR) in the germinal centre to subsequently become either 2) long-lived plasma cells, which home to the bone marrow, or 3) long-lived memory B cells ^1– 4^.

Human memory B cells were originally isolated based on their lack of IgD expression, which had been identified as a naïve B cell marker ^5, 6^. Subsequent to this, two papers identified CD27 as a general marker of B cell memory ^7, 8^. This newly identified memory B cell marker allowed for a more refined study of the bulk memory B cell population. However, the CD27 ^+^ population is heterogeneous and is comprised of roughly 10–20% IgM ^+^ IgD ^+^, 40–50% IgM ^+^ IgD ^-^ and 30–40% IgM ^-^ IgD ^-^ isotype switched cells. The existence of IgM ^+^ CD27 ^+^ cells as T cell-dependent memory B cells has been hotly debated ^9^. Nonetheless these IgM ^+^ memory B cells do show classical memory cell hallmarks, such as somatically hypermutated V genes ^8, 10^, and a recent in depth study of this population has shown that they participate in T cell-dependent recall responses and show similar transcriptome patterns to the IgM ^-^ IgD ^-^ CD27 ^+^ population ^11^. Therefore, to gain a more complete understanding of the memory B cell response, it will be important to delineate the functionality of these T cell-dependent memory B cell subsets.

Memory B cells are central players of long-term humoral immunity, capable of responding rapidly and with high affinity to secondary encounter with an antigen. Successful vaccination readily induces long-lived B cell memory that is maintained for decades ^12, 13^. Recent observations have shown that vaccination or infection does not, however, produce a homogenous population of memory B cells, but a constellation of subsets depending on the kinetic time point, location, and type of vaccination or infection ^14, 15^. The frequency of memory B cell subsets is variable, with some subsets such as immunoglobulin (Ig)D ^+^ IgM ^-^ CD27 ^+^ memory B cells forming only 1–3% of peripheral blood B cells ^14^, and this number could be even smaller when looking at vaccine induced antigen specific responses. Despite their rarity, such subsets could play an important role in the immune response to infection and/or vaccination. For instance, IgM ^+^ memory B cells have recently been shown to play an important role in the early response to malaria re-challenge using a murine model ^16^, whilst human IgM ^+^ memory B cells have been shown to play a role in decreasing Rotavirus viral load ^17^.

A number of different assays have been developed to facilitate the investigation of the memory B cell repertoire in response to vaccination or infection. The use of fluorophore-tagged antigen to identify antigen-specific memory B cells has been attempted with some success ^18– 20^. However, optimisation of antigen-specific B cell staining is a complex process and carries a number of potential pitfalls. Three of the major issues with antigen-specific staining are the scarcity of the cells, the low levels of surface Ig expression and the need for a highly purified antigen, which can make identification of antigen specific B cells difficult ^21^. In an effort to avoid these issues, Epstein-Barr virus immortalisation of memory B cells followed by screening of cell culture supernatant for antigen reactivity has been performed. This technique, however, has its own limitations, such as immortalisation biases and low immortalisation efficiency ^22, 23^. A more recent transformation based approach utilises a retroviral transduction system to induce expression of the antiapoptotic factors Bcl-6 and Bcl-xL, which, when combined with IL-21 and CD154 allows memory B cells to differentiate into long lived antibody secreting cells (ASCs) that still retain surface BCR expression ^24, 25^.

*Ex vivo* expansion and differentiation of memory B cells into ASCs is an alternative technique that has now been widely adopted in the field, owing to its simplicity and versatility. This technique allows a variety of different functional assays to be undertaken allowing for a more complete interrogation of the memory B cell repertoire. ELISA and ELISpot assays can quantify antigen-specific Ig and define the Ig isotype secreted by the expanded memory B cells, viral neutralisation assays assess the functionality of the antibody, and bio-layer interferometry permits measurement of the antibody binding kinetics. For example, *ex vivo* memory B cell expansion has been recently used to identify an extremely potent HIV-1 broadly neutralising antibody named N6, which could not be identified through flow cytometry based approaches ^26^. Overall these downstream assays can be applied to answer a number of important biological questions. For example, investigating the magnitude of the memory B cell subset response to vaccination or infection, the reactivity of the recall response between different memory B cell subsets and mapping the specificity of the response and how this evolves between different memory B cell subsets ^26^.

To date, a plethora of different conditions capable of inducing memory B cell expansion/differentiation have been published. Combinations of cytokines, such as IL-2, IL-10, IL-21 ^27– 33^, pattern recognition receptor agonists such as R848, CpG ODN _2006_^28, 30, 34^ and CD40 stimulation ^35^, form the basis of most published conditions. In 2009, Pinna *et al.* ^30^ published one of the most widely utilised methodologies owing to its simplicity and robust expansion capability. This methodology consisted of the addition of IL-2 and R848 to isolated B cells, with irradiated peripheral blood mononuclear cells (PBMCs) acting as the CD154 (CD40-ligand) source. However, despite detailed analysis of the origins of memory B cell subsets ^36^ and optimisation of *ex vivo* memory B cell culture conditions for the investigation of the IgG ^+^ response ^37^, no conditions to date have been investigated for their ability to induce maximal and proportional memory B cell expansion/differentiation across the CD27 ^+^ IgM ^-^ IgD ^-^, IgM ^+^ IgD ^+^ and IgM ^+^ IgD ^-^ subsets. Defining such conditions will be important in allowing a comprehensive assessment of how the memory B cell response evolves between these subsets across time in response to infection and/or vaccination. Identification of these conditions will also have implications for the study of rare polyreactive memory B cells which are difficult to fully investigate using conventional fluorophore tagged antigen approaches. By inducing expansion and differentiation of single memory B cells, including the IgM ^+^ subsets, the culture supernatants could easily be screened for reactivity to multiple antigens.

 In this study, we screened a wide variety of published memory B cell expansion stimuli and then utilised a Design of Experiments (DoE) approach to identify the optimal combination across different CD27 ^+^ memory B cell subsets. The expansion and differentiation of memory B cells to ASCs was then tracked via flow cytometry and IgH deep sequencing. 

## Methods

### PBMC and memory B cell isolation

Written informed consent was obtained from all 10 donors. All samples were collected under protocols approved by the Imperial College NHS Trust Tissue Bank and the National Research Ethics Committee in accordance with the Human Tissue Act 2004. Approval for this project was granted by the Imperial College Healthcare Tissue Bank, under their HTA research licence, and ethics thus conveyed through this process by the Multi Research Ethics Committee (MREC), Wales. PBMCs were isolated by centrifugation (400 × g, 30 min, no brake) over Histopaque-1077 (Sigma Aldrich, Dorset, UK). CD27 ^+^ memory B cells were then isolated using the Memory B Cell Isolation Kit (Miltenyi Biotec, Surrey, UK) following the manufacturer’s instructions. Due to the rarity of some subsets the same donors could not be used throughout the whole study. Therefore, memory B cells were isolated from 10 different donors and replicates from 1–3 donors used per individual experiment. This meant that inter donor variability was measured throughout each experiment but not between different experiments. However, it should be noted that all isolated memory B cells and subsets from all donors were well within the expected normal range.

### Literature review

In order to identify stimuli associated with current memory B cell culture protocols, a literature review was carried out using the following search terms: memory B cell ELISpot, memory B cell culture, memory B cell stimulation, memory B cell differentiation and memory B cell expansion using the NCBI PubMed database ( https://www.ncbi.nlm.nih.gov/pubmed). The results of this literature review can be seen in Table 1.

**Table 1.** Expansion stimuli including the concentrations used in both the original screening process and the Design of Experiments (DoE) process. Concentrations were chosen to reflect those shown in the literature. APRIL concentrations were chosen to mirror that of BAFF, as APRIL has not been previously published as a stimulus for inducing memory B cell differentiation.

Expansion factor	Original screen concentrations	Reference	DoE concentrations	Target
IL-2	100, 500, 1000 U/ml	30, 32	N/A	IL-2R
IL-6	10, 50, 100 ng/ml	31, 32	N/A	IL-6R
IL-15	10, 50, 100 ng/ml	32, 38, 39	N/A	IL-15R
IL-21	10, 50, 100 ng/ml	28, 31	10, 50, 100 ng/ml	IL-21R
BAFF	10, 50, 100 ng/ml	27, 38	N/A	BAFF-R, BCMA, TACI
APRIL	10, 50, 100 ng/ml	32	N/A	TACI, BCMA
CpG ODN _2006_	0.5, 2.5, 10 μg/ml	12, 28, 34, 40	0, 0.25, 1 (g/ml	TLR9
PWM	5, 50, 100 ng/ml	28, 34, 41	N/A	TLR2 or indirectly via T cells
R848	0.5, 1, 5 μg/ml	30, 42	0, 0.25, 0.5 (g/ml	TLR7/TLR8
HV13280 cells	Utilised at a ratio of 1:4 with memory B cells		1:5, 1:2, 1:1	CD40

### Cell culture and stimulation conditions

RPMI-1640 media (Sigma Aldrich) supplemented with L-glutamine, Penicillin/Streptomycin and 10% fetal bovine serum (FBS) (Sigma Aldrich) was used throughout the study. Isolated memory B cells were set at different cell densities in 96-well U-bottom plates (2×10 ^3^ cells/well in 250 μl) or in 24-well flat bottom plates (1×10 ^6^ cells/well in 1 ml). Small-scale expansions were used for sequencing and Ig quantification, large-scale expansions were used for phenotyping of expanding cells by flow cytometry. Before adding memory B cells, each well was seeded with irradiated (2,000 cGray) HV13280 feeder cells (CD154 ^+^ HEK-293T cells, kindly provided by L. Liao’s lab, Duke University, Durham, NC, USA) at ratios varying from 1:1 to 1:50 (HV13280:memory B cell). After the addition of memory B cells, cultures were stimulated with iterative combinations of the following stimuli: recombinant human interleukin (IL)-2 (100–1000 U/ml), IL-6 (10–100 ng/ml), IL-15 (10–100 ng/ml), IL-21 (10–100 ng/ml), APRIL (10–100 ng/ml), BAFF (10–100 ng/ml), CpG ODN _2006_ (0.25–10 μg/ml) (HyCult Biotech, Uden, Netherlands); R848 (0.25–5 μg/ml; Invivogen, Toulouse, France); PWM (5–100 ng/ml; Sigma Aldrich). Cells were then cultured for 5 or 10 days at 37°C 5% CO _2_. All recombinant proteins were ordered from Peprotech (London, UK) unless otherwise stated.

### ELISA

Total IgG, IgA and IgM in culture supernatants were measured by ELISA. Nunc MaxiSorp 96 well plates were coated overnight at 4°C with 100 μl goat anti-human kappa/lambda (Southern Biotech, Cambridge, UK; product number: 2060-01/2070-01) diluted 1:500 in PBS. Plates were washed with PBS/0.05% Tween-20 and blocked with 200 μl PBS/0.05% Tween-20/1% bovine serum albumin (BSA) (Sigma Aldrich) for 1 hour at 37°C. Plates were then washed and 50 μl of culture supernatant diluted in blocking buffer added to each well and incubated for 1 hour at 37°C. Following incubation and washing, 100 μl of detection antibody diluted in blocking buffer was added: goat anti-human peroxidase IgG (1:20,000), IgA (1:10,000), and IgM (1:1,000) (Sigma Aldrich; product numbers: A0170, A0295, and A6907, respectively). Plates were washed and developed using TMB (KPL, Middlesex, UK), stopped using 1% HCl stopping solution (KPL) and read using the VersaMax microplate reader (Molecular Devices, Berkshire, UK).

### Flow cytometry and cell sorting

Memory B cells were stained with 5 μM Cell Trace Violet (ThermoFisher Scientific, Paisley, UK) as directed in the datasheet, and incubated overnight at 37°C 5% CO _2_. The cells were then washed and cultured using the optimal DoE conditions. For flow cytometry experiments cells were then stained with Aqua Live/Dead cell viability dye (BD Biosciences, Oxford, UK) as per manufacturer’s instruction. Cells were then stained with the phenotyping panel shown in Table 2. The cells were analysed using an LSR Fortessa II cytometer (BD Biosciences) at baseline, day 5 and day 10 of culture. The gating strategy used can be seen in Supplementary Figure 1. Purity of CD27 ^+^ memory B cells following isolation by magnetic selection was also determined using this panel. For FACS, memory B cells were sorted based upon IgD and IgM expression into 4 sub-populations (IgD ^+^ IgM ^-^, IgD ^+^ IgM ^+^, IgD ^-^ IgM ^+^, IgD ^-^ IgM ^-^) as shown in Supplementary Figure 1. Cell sorting was carried out using a BD FACSAria III.

**Table 2.** B cell phenotyping flow cytometry panel. Volumes shown represent the staining volumes used, topped up to 100 μl with FACS buffer (1×PBS, 25mM Hepes [Sigma Aldrich], 1mM EDTA [Sigma Aldrich], 2.5% FBS), per 1×10 ^6^ cells. Volumes used were titrated in-house.

Marker	Fluorophore	Channel	Supplier	Clone	Isotype	Volume
CD3	V500	405-525/50	BD: 561416	UCHT1	Mouse IgG1 κ	1.25μl
CD4	V500	405-525/50	BD: 560768	RPA-T4	Mouse IgG1 κ	1.25μl
CD14	V500	405-525/50	BD: 561391	M5E2	Mouse IgG2a κ	2.5μl
CD19	BV605	405-605/12	Biolegend (London, UK): 363024	SJ25C1	Mouse IgG1 κ	1.25μl
CD27	PE Cy7	561-780/60	Biolegend: 356412	M-T271	Mouse IgG1 κ	2.5μl
CD38	APC	640-670/14	Biolegend: 356606	HB-7	Mouse IgG1	0.6μl
CXCR4	PE	561-582/15	Biolegend: 306506	12G5	Mouse IgG2a κ	0.6μl
IgM	FITC	488-530/30	Biolegend: 314506	MHM-88	Mouse IgG1 κ	1.25μl
IgD	PE-CF594	561-610/20	BD: 562540	IA6-2	Mouse IgG2a κ	1.25μl
IgG	APC-H7	640-780/60	BD: 561297	G18-145	Mouse IgG1 κ	1.25μl

### Library preparation for next generation sequencing

Following cell sorting, the four memory B cell subsets were cultured using the optimal DoE expansion conditions with cells removed and IgH sequencing carried out at baseline, day 5 and 10 of culture. RNA extraction was performed using RNeasy Micro Kit (Qiagen, Manchester, UK) according to manufacturer’s protocol. Reverse transcription (RT) was run as a 20 μl reaction with SuperScript® III (Thermo Fisher, Loughborough, UK). cDNA was cleaned-up with Agencourt AMPure XP beads (Beckman Coulter, Buckinghamshire, UK). Reagents for each RT step were divided in two mixes: Mix1 (RNA template, barcoded multiplex Constant region primer set [10 μM each primer], nuclease-free water) was incubated for 1 min at 70°C and then immediately transferred on ice for 1 min; Mix2 (4 μl 5x FS buffer, 1 μl DTT [0.1 M], 1 μl dNTP [10mM], 1 μl SuperscriptIII) was added and incubated at 50°C for 60 min followed by inactivation at 70°C for 15 min. Cleaned cDNA was amplified with V-gene multiplex primer mix (10 μM each forward primer) and 3’ universal reverse primer (10 μM) using KAPA Real-Time Library Amplification Kit (KAPA Biosystems, Wilmington, MA, USA) under the following thermal cycling conditions: 1 step (95°C - 5 min); 5 cycles (98°C - 5 sec; 72°C - 2 min); 5 cycles (65°C - 10 sec, 72°C - 2 min); 25 cycles (98°C - 20sec, 60°C - 1 min, 72°C - 2 min); 1 step (72°C - 10 min). Nucleotide sequences for primers can be seen in Table 3.

**Table 3.** Primer sequences.

Primer Name	Primer Sequence	IgH Binding Region
IGHA	TGTCCAGCACGCTTCAGGCTNNNNTNNNNTNNNNGAYGACCACGTTCCCATCT	C region
IGHM	TGTCCAGCACGCTTCAGGCTNNNNTNNNNTNNNNTCGTATCCGACGGGGAATTC	C region
IGHD	TGTCCAGCACGCTTCAGGCTNNNNTNNNNTNNNNGGGCTGTTATCCTTTGGGTG	C region
IGHE	TGTCCAGCACGCTTCAGGCTNNNNTNNNNTNNNNAGAGTCACGGAGGTGGCATT	C region
IGHG	TGTCCAGCACGCTTCAGGCTNNNNTNNNNTNNNNAGTAGTCCTTGACCAGGCAG	C region
VH1-FR1	GGCCTCAGTGAAGGTCTCCTGCAAG	V region
VH2-FR1	GTCTGGTCCTACGCTGGTGAAACCC	V region
VH3-FR1	CTGGGGGGTCCCTGAGACTCTCCTG	V region
VH4-FR1	CTTCGGAGACCCTGTCCCTCACCTG	V region
VH5-FR1	CGGGGAGTCTCTGAACATCTCCTGT	V region
VH6-FR1	TCGCAGACCCTCTCACTCACCTGTG	V region
3'universal	TGUCCAGCACGCTUCAGGC	n/a

### Next generation sequencing and barcode filtering

MiSeq libraries were prepared using Illumina protocols and sequenced using 300bp paired-ended MiSeq (Illumina, Cambridge, UK). Raw MiSeq reads were filtered for base quality (median Phred score >34) using the QUASR program version 6.08 ( http://sourceforge.net/projects/quasr/ ^43^) MiSeq forward and reverse reads were merged together if they contained identical overlapping region of >50bp, or otherwise discarded. Universal barcoded regions were identified in reads and orientated to read from V-primer → constant region primer. The barcoded region within each primer was identified and checked for conserved bases (i.e. the T’s in NNNNTNNNNTNNNNT). The reads were checked for homology to the first 50bp of the reference constant region genes from the IMGT database ( http://www.imgt.org/vquest/refseqh.html) ^44^ by k-mer matching (where k=10bp). The closest matching constant region allele was identified, and information retained throughout the analysis. Primers and constant regions were trimmed from each sequence, and sequences were retained only if there was >80% sequence certainty between all sequences obtained with the same barcode, otherwise discarded. Sequences without complete reading frames and non-immunoglobulin sequences were removed and only reads with significant similarity to reference IgHV and J genes from the IMGT database were retained using BLAST ^45^.

### Analysis of VH SHM, CDRH3 length and isotype-distribution of BCR repertoires

Isotype information was derived from constant region assignment of each BCR read according to IMGT. Isotype structure of each sorted B cell population across the three time points (baseline, day 5 and day 10) was calculated as percentage of reads from a given sample, assigned to each isotype. SHM levels and CDRH3 length was determined using IMGT-HighV-Quest (version 1.5.0) ( https://www.imgt.org/HighV-QUEST/).

### Design of experiments approach

For the DoE approach, we utilised a full factorial design where each of the four chosen stimuli (IL-21, HV13280 cells, CpG ODN _2006_ and R848) would be tested for their effect on Ig secretion as measured by ELISA, at three different chosen concentrations, generating a total of 3 ^4^ = 81 different possible conditions. The first order and second order sensitivity indices reflecting the effect of each stimuli on Ig secretion and the p-values (shown to 4 decimal places) were then determined using a custom MATLAB script based on the use of an N-way ANOVA (see Data availability).

### Statistics

Statistical tests were performed in MATLAB (Version 2014; MathWorks, Natick, MA, USA) or in Prism (Version 7; GraphPad, San Diego, CA, USA). For the final comparison set, two-way analysis of variance (ANOVA) with Tukey’s post-hoc test was used for statistical analysis.

## Results

### Memory B cells isolated from PBMC are efficiently differentiated into high Ig secretory ASC by culture with optimal levels of IL-21, TLR and CD40 co-stimulation

In order to identify the conditions best suited for inducing memory B cell expansion and differentiation towards ASCs, a literature review of suggested culture conditions was carried out ( Table 1). Following identification of a wide range of conditions used for the induction of memory B cell differentiation, a checkerboard approach combining any two suggested factors with memory B cells and HV13280 cells (CD154 ^+^ feeder cell line) was set up. Each two-way crossover was carried out with factors at one of three chosen concentrations, these concentrations were chosen to be reflective of the publications that they were obtained from. All possible crossovers of the listed factors were carried out except for conditions combining two pattern recognition receptor (PRR) agonists, such as R848 and Pokeweed mitogen (PWM). CD40 stimulations were used for all checkerboard expansions as they were the one constant used throughout the published differentiation conditions and thus seen to be essential.

**Figure 1.**
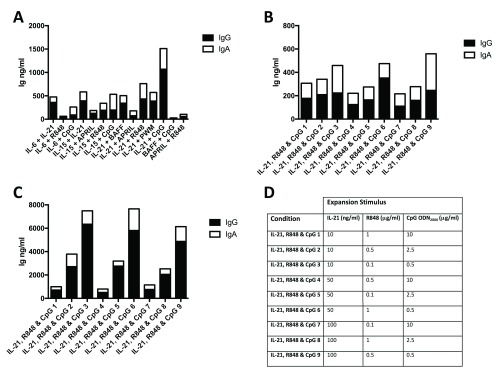
CD40 stimulation in combination with IL-21 & TLR stimulation of memory B cells over a 10 day culture period induces high levels of Ig secretion. ( **A**) 2×10 ^3^ memory B cells were cultured for 5 days with HV13280 cells at a ratio of 4:1 with the addition of a checkerboard of different stimulants suggested to play a role in memory B cell expansion/differentiation (see Table 1). Total Ig (IgG + IgA) was measured in the culture supernatant by ELISA and the top readout for each crossover is shown. The full checkerboard screen can be seen in Supplementary Figure 2. ( **B** and **C**) Total Ig (IgG + IgA) measured by ELISA in culture supernatant of memory B cells stimulated with HV13280 cells, IL-21, R848 and CpG at ( **B**) day 5 and ( **C**) day 10 of culture. ( **D**) Composition of the stimulation mixtures used in B & C. Data is representative of one donor.

After 5 days of culture, total Ig (IgG + IgA) in the culture supernatants was measured by ELISA. Ig in the supernatant acts as a readout for memory B cell differentiation as the memory B cells differentiate from being surface Ig-expressing cells to Ig-secreting cells. The results suggested that a combination of either IL-21 and CpG or IL-21 and R848 induced the highest levels of Ig secretion ( Figure 1A, Supplementary Figure 2). These results matched a previously published trend ^28^.

In an attempt to drive further differentiation and expansion, combinations of the CD40 stimulation, IL-21, R848 and CpG were assessed ( Figures 1B–D), with memory B cells cultured for 5 ( Figure 1B) or 10 days ( Figure 1C). For these expansions the R848 concentrations were lowered in an attempt to prevent over-stimulation when combining TLR agonists. This final screen identified that a 10-day culture period induced higher levels of memory B cell differentiation and that there was a trend for higher Ig secretion with lower levels of CpG ODN _2006_ and R848 combined.

### A full factorial DoE approach identified significant individual effects of IL-21, R848 and CD154 on Ig secretion from memory B cells

Following the identification of CD40 stimulation, IL-21, R848 and CpG as having the greatest capacity for inducing memory B cell expansion/differentiation over a 10-day culture period, these expansion stimuli were combined in a targeted approach that would allow their individual effects on memory B cell differentiation to be titrated. We utilised a full factorial DoE approach with four expansion stimuli at three set levels (low/intermediate/high) ( Table 1), generating 81 possible combinations (3 ^4^). The concentrations chosen for this approach were based on the trends observed in data presented in Figure 1. 2×10 ^3^ memory B cells from three individual donors were cultured in triplicate with each possible combination over a 10-day culture period and total Ig (IgG, IgM & IgA) levels in the supernatant measured.

**Figure 2.**
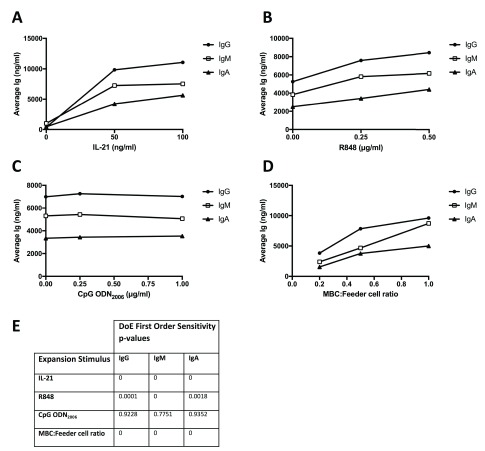
A Design of Experiments approach identifies IL-21, CpG and CD40 stimulation as having significant first order effects on IgG, IgM & IgA secretion by differentiated memory B cells. 2×10 ^3^ memory B cells were cultured with all possible combinations of HV13280 cells, IL-21, R848 and CpG at three set concentrations for 10 days, after which total Ig (IgG, IgA & IgM) was measured in culture supernatant by ELISA. ( **A**– **D**) Using a Matlab script, the effect of ( **A**) IL-21, ( **B**) R848, ( **C**) CpG and ( **D**) CD40 stimulation at the chosen concentrations on IgG, IgM and IgA secretion could be determined. ( **E**) P-values showing the effects of each expansion stimulus on IgG, IgM and IgA secretion into culture supernatant over the 10 day culture. Data shows a summary of three independent donors.

A DoE full factorial approach allows for the impact on Ig output to be determined for each stimulant, at each set concentration ( Figures 2A–E, Supplementary Figure 3). Through the use of a MATLAB script the mean Ig detected in the supernatant whilst “variable x” remains constant and all other variables altered can be calculated, ultimately allowing the impact of subsequently changing the concentration of “variable x” on Ig secretion to be measured. Subsequently 2 ^nd^ order interactions and their significance can also be assessed, as there are 9 combinations per donor where two stimulants will remain at the same concentration whilst all other stimulants are being altered.

**Figure 3.**
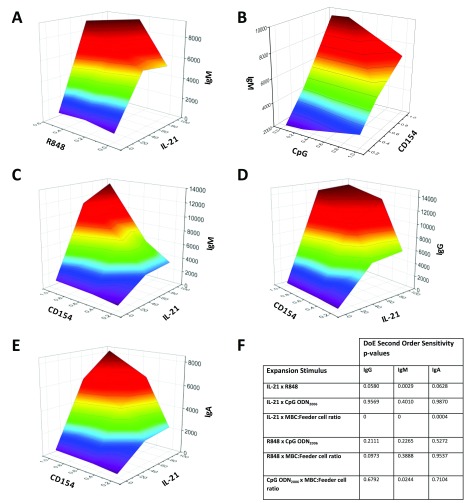
A Design of Experiments approach identifies IL-21 & CD40 stimulation as having the greatest second order interaction effect on IgM, IgG & IgA secretion by differentiated memory B cells. ( **A**– **E**) Colour plots of the significant second order interactions showing their effect on IgM, IgG and IgA secretion. The non-significant second order interaction colour plots can be seen in Supplementary Figure 5. ( **F**) P values of the second order interactions on IgG, IgM and IgA secretion into culture supernatant over the 10 day culture. Data shows a summary of three independent donors.

We determined that a combination of high IL-21 ( Figure 2A), high R848 ( Figure 2B) and high CD40 stimulation ( Figure 2D) induced the highest levels of Ig secretion, whilst all having a significant first order impact on Ig secretion ( Figure 2E). Importantly, no stimulant appeared to bias the induction of secretion of one isotype over another. As well as having significant first order effects, the combination of IL-21 and CD40 stimulation had a significant second order impact on IgG, IgM & IgA secretion ( Figures 3C–F), whilst R848 in combination with IL-21 and CpG in combination with CD40 stimulation had significant effects on IgM secretion ( Figures 3A and B). All other second order interactions were not significant ( Supplementary Figure 4). Of note was the determination that CpG did not impact Ig secretion ( Figure 2C), with total Ig in culture supernatant remaining the same when CpG was at 0, 0.25 or 1 μg/ml. Therefore, we defined the optimal expansion conditions as 1:1 MBC:HV13280 ratio, 100 ng/ml IL-21, and 0.5 μg/ml R848.

**Figure 4.**
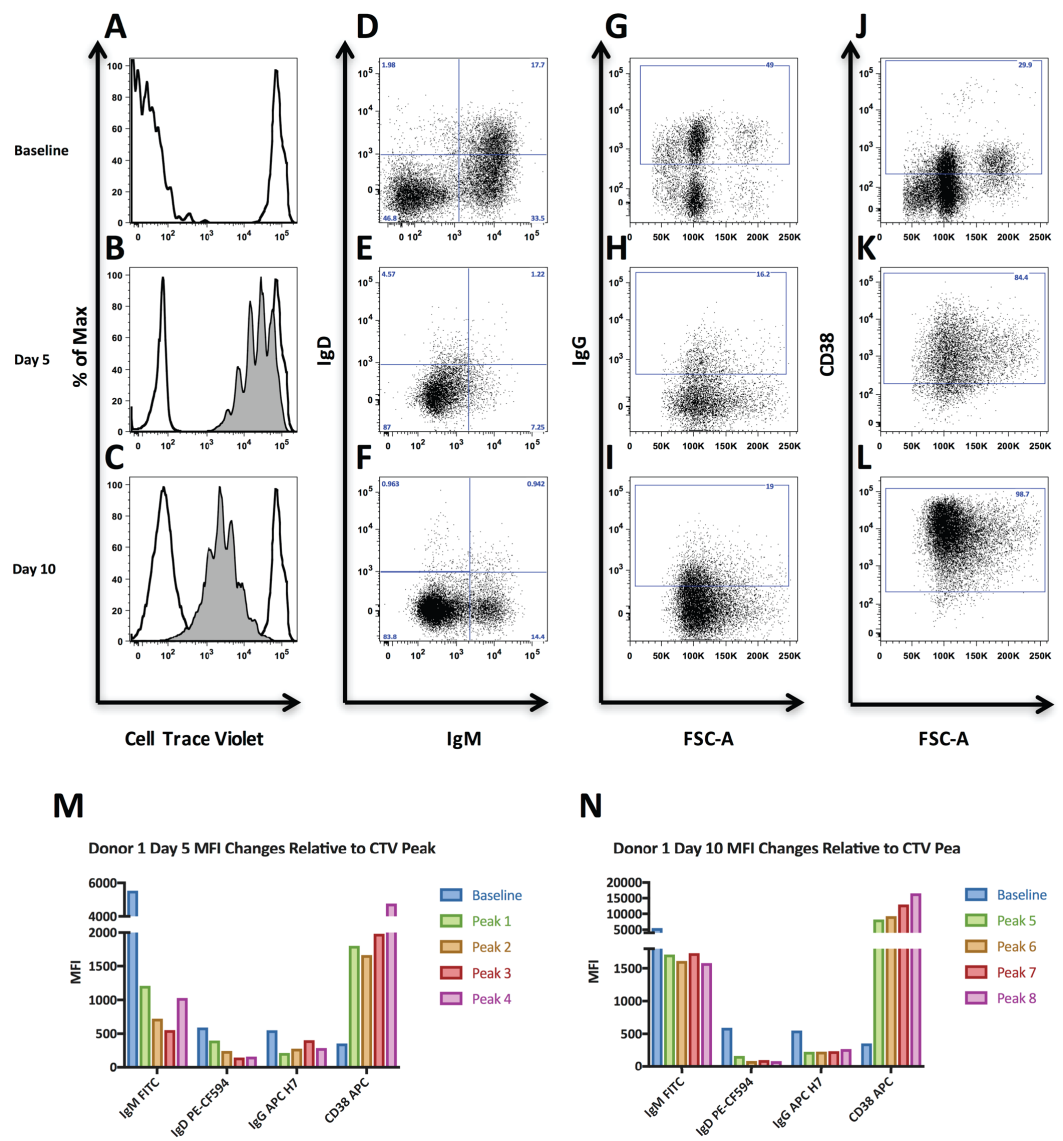
Upon stimulation with the optimal Design of Experiments (DoE) expansion conditions, memory B cells progressively differentiate into antibody secreting cells. Cells were stained with cell trace violet and cultured with the optimised DoE conditions over 10 days, CD19 ^+^ CD27 ^+^ lymphocytes were phenotyped at baseline, day 5 and day 10. Baseline, day 5 and day 10 ( **A**– **C**) cell trace violet levels, ( **D**– **F**) surface IgD and IgM expression, ( **G**– **I**) surface IgG expression and ( **J**– **L**) CD38 expression. ( **M** and **N**) Mean fluorescence intensity (MFI) values for each fluorophore relative to the cell trace violet (CTV) peak. A minimum of 5,000 cell trace violet positive events were acquired for each time-point. Data is representative of two independent donors.

We subsequently compared total Ig (IgG, IgM & IgA) secretion induced by the DoE conditions to a literature comparator (IL-2 plus R848) ^30^ and IL-21 plus CpG (optimal condition in the original 2 parameter screen). Memory B cells were stimulated with a number of the top DoE conditions, IL-2 and R848 used at concentrations selected to reflect those detailed in the literature or IL-21 and CpG at concentrations to mirror those in the original screen ( Supplementary Figure 5B). HV13280 cell:memory B cell ratios of 1:1 identified by the DoE process as optimal were used throughout for IL-2 plus R848 and IL-21 plus CpG.

The results showed that the identified DoE conditions significantly induced (p=0.0003) higher Ig secretion levels than IL-2 and R848 ( Supplementary Figures 5A and C). The optimal condition also induced significantly higher levels of Ig secretion than the majority of IL-21 and CpG combinations. However, although the top DoE condition induced higher levels of Ig secretion than IL-21 and CpG combination 6, the difference was not significant.

### Memory B cells differentiate into plasmablast-like cells upon stimulation with optimal DoE conditions

Upon identification of the optimal memory B cell differentiation conditions through the use of a full factorial DoE approach, the differentiation of memory B cells over a 10-day culture period was tracked by flow cytometry. Freshly isolated CD27 ^+^ memory B cells were labelled with the cell tracking dye CellTrace™ violet, and put into culture with the optimal DoE expansion/differentiation conditions. Cells were phenotyped at baseline, day 5 and day 10 of culture. CellTrace™ violet identified multiple rounds of cell division by the end of day 10 ( Figures 4A–C). Additionally, cells progressively lost expression of surface Ig, as demonstrated by a loss in IgD and IgM staining ( Figures 4D–F). The loss of surface Ig was further confirmed by a coincident loss in IgG staining ( Figures 4G–I). This result confirmed that the culture conditions were not inducing class switching of previously IgD and/or IgM positive cells to double negatives, but were rather causing the loss of surface Ig expression. Finally an increase in CD38 expression was also detected ( Figures 4J–L). Increased CD38 expression was particularly telling as it is a B cell marker routinely used for the identification of ASCs, such as plasmablasts and plasma cells ^46, 47^. Interestingly although the surface Ig expression was rapidly decreased, CD38 expression levels changed relative to the number of divisions that had taken place ( Figures 4M and N). This suggests that as the cells proliferate they progressively differentiate towards ASCs.

 Ultimately, by the end of the 10-day culture period, CD27 ^+^ memory B cells had undergone multiple rounds of cell division, differentiating from CD38 ^-^, surface Ig expressing cells, to CD38 ^+^ plasmablast-like cells that had lost the majority of their surface Ig expression. These phenotypical changes coincide with the ability of the cells to secrete Ig, as was detected in culture supernatant by ELISA. Therefore, the optimised DoE expansion conditions promote the phenotypic and functional differentiation of memory B cells into ASCs.

### The DoE optimised memory B cell expansion/differentiation conditions do not induce Ig locus genotypic changes

**Figure 5.**
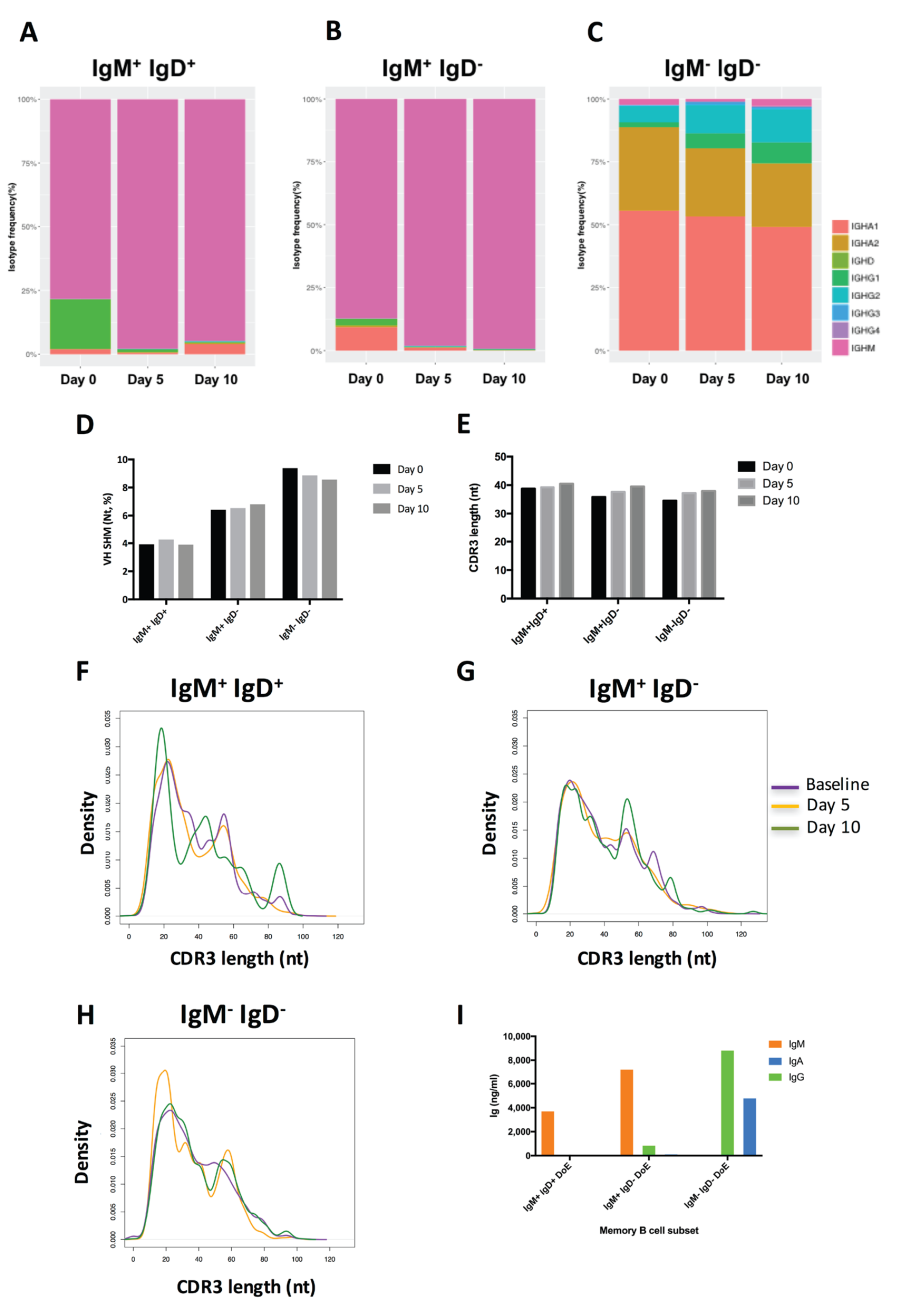
Design of Experiments expansion/differentiation conditions do not induce undesirable Ig genotypic changes. IgM ^+^ IgD ^+^, IgM ^+^ IgD ^-^, IgM ^-^ IgD ^-^ memory B cells were isolated by cell sorting and cultured using the optimised conditions. Samples were taken at baseline, day 5 and day 10 where Ig transcripts were analysed. ( **A**– **C**) Ig constant region transcripts measured from each subset at baseline, day 5 and day 10 of culture expressed as a percentage of isotype-specific Ig reads from the total Ig repertoire. ( **D**) VH SHM of each memory B cell subset across the three timepoints as a percentage of the total VH segment length at nucleotide level. ( **H**) Average CDRH3 length in nucleotides of Ig transcripts measured for each population across the different time points. ( **F**– **H**) Density plots of CDRH3 length for the individual subsets across the three time points. CDRH3 length is shown as nucleotides on the x axis. ( **I**) IgG, IgA and IgM levels in culture supernatant when memory B cell subsets are cultured separately. Data is representative of two independent donors.

One of the key issues with *ex vivo* proliferation and differentiation of memory B cells is ensuring that undesirable Ig locus changes that could alter the reactivity of the secreted immunoglobulin are not induced. To detect such changes, we employed a sequencing approach where we first sorted each memory B cell subset (IgM ^+^IgD ^+^, IgM ^+^IgD ^-^ and IgM ^-^IgD ^-^) having retained a sample for baseline reads and then cultured for 5 and 10 days before next generation sequencing of the total population Ig transcripts. We first looked at CSR events, and the data show that the IgM ^+^IgD ^+^ and IgM ^+^IgD ^-^ subsets remain largely IgM ^+^ throughout the culture period ( Figures 5A, B and I, Supplementary Figures 6A and B). Whilst the IgM ^-^ IgD ^-^ population showed a distribution of different IgG and IgA isotypes, which largely remained within their relative proportions over the 10-day culture period ( Figures 5C and I, Supplementary Figure 6C). Overall these data suggest that the culture conditions do not induce CSR events both at the RNA and protein level.

To assess levels of SHM, we first looked at how the level of VH SHM changed across the 10 day culture period for each subset ( Figure 5D). This data showed that the level of SHM did not increase across the 10 day culture period. Subsequently, we investigated how CDRH3 length changed across the 10 day culture period. The results show that within each subset the average CDRH3 length remains almost constant in terms of nucleotide length ( Figure 5E) across the 10-day culture period, with a more detailed analysis showing that for each subset the densities of the different CDRH3 lengths present in the population appear to remain constant across the different time points ( Figures 5F–H, Supplementary Figures 6E–G). Overall this data suggests that although the culture conditions were inducing proliferation and differentiation of memory B cells they were not inducing SHM events.

## Discussion

The role of memory B cell subsets in response to vaccination and/or infection is only just starting to become clear. Differences in location, kinetic time point and type of vaccination or infection all influence the development of different memory B cell subsets. Importantly it is not just phenotype that distinguishes these cells from one another, they may each have differences in functionality ^11^, switch capacity ^48^ and overall numbers in responses to different types of challenge.

To effectively study memory B cell subsets, we carried out a wide screen of memory B cell expansion and differentiation conditions and utilised a novel DoE process to optimise conditions that would lead to the highest levels of proliferation and Ig secretion. Subsequently the optimal conditions were characterised to ensure that they did not induce any undesirable changes.

Our original wide screening process encapsulated over 200 different culture conditions taken from the literature. The results from this section clearly demonstrated a trend towards higher Ig secretion in those wells where CD40 stimulation was combined with IL-21 and some form of PRR agonist. The use of IL-21 for *ex vivo* memory B cell proliferation and differentiation stems from its essential role in the germinal center ^49^ where it acts directly on B cells. Therefore, IL-21 serves an essential role in several different published protocols for both human and murine memory B cell culture ^26, 29, 50^. The combination of CD40 stimulation, IL-21 and CpG for memory B cell culture has been previously published ^28^. However, what had not been attempted was the combination of CD40 stimulation, IL-21, CpG and R848, which formed the basis of the top two conditions identified from our primary screen.

To try and induce maximal memory B cell proliferation and differentiation, we combined the top two conditions from our original screen using a structured DoE full factorial design. The DoE full factorial design approach offers a powerful statistical tool to answering biological questions and can ultimately save time and costs, and streamline the addressing of research questions from *in vitro* work through to *in vivo*, as reviewed by Shaw *et al.* ^51^. This approach suited our needs, as it allowed us to determine the impact of each stimulant on Ig secretion. A total of 81 possible combinations (3 concentrations of 4 different stimulants) were assessed. The data showed that a combination of 100 ng/ml IL-21, 0.5 μg/ml R848 and high levels of CD40 stimulation all significantly enhanced Ig secretion. The fact that CpG did not significantly enhance Ig secretion was surprising, particularly as the CD154, IL-21, CpG condition induced higher levels of Ig than CD154, IL-21 and R848 in the original screen. The explanation for this likely lies in the fact that the concentrations used in the DoE approach were chosen based on trends seen in the original screen. The lack of an effect then witnessed by CpG was possibly due to a redundancy mechanism, whereby the R848 signal that was now at a more optimal concentration than in the original screen outcompeted the CpG, as both R848 and CpG use TLRs which signal through the MyD88 adaptor protein ^52^. Also of note from the DoE data is that although we observed a potential plateau in Ig secretion with increasing concentrations of CD40 stimulation, IL-21 and R848 responses were still trending upwards. Therefore, it is possible that higher concentrations of each of these stimulants could further enhance expansion and Ig secretion. Finally, and perhaps most importantly, the DoE approach was capable of showing that neither IL-21, R848 nor CD40 stimulation at any concentration appeared to bias Ig secretion towards either IgG, IgA or IgM. This suggested that the optimal conditions were not biased towards inducing proliferation and or differentiation of either IgM ^+^ IgD ^+^, IgM ^+^ IgD ^-^, IgM ^-^ IgD ^-^ memory B cell subsets.

To confirm if the DoE process was beneficial, we went on to compare the Ig secretion induced by IL-2 & R848, IL-21 & CpG from our original screen, and the DoE optimised conditions. Overall the results justified the DoE optimisation approach. However, the results did show that although inducing significantly higher levels of Ig secretion than IL-2 and R848, the difference between the DoE conditions and the IL-21 & CpG conditions was not always significant, despite a trend towards higher Ig secretion with the DoE conditions. The lack of an effect seen with the IL-2 and R848 was rather surprising. The original publication on the optimisation of the IL-2 and R848 conditions by Pinna *et al.* ^30^ was for memory B cells within a PBMC scaffold, and the authors also showed that increased levels of CD40 stimulation inhibit memory B cell proliferation. Therefore, it is possible that our CD154 levels that were optimised for the IL-21 conditions could have been inhibitory in the IL-2 and R848 cultures. 

To characterise how the memory B cell culture conditions impacted the Ig locus, we employed an IgH deep sequencing platform. One issue we faced in our sequencing work was the presence of IgG1 and IgA1 transcripts detected at baseline in the IgM ^+^ subsets. This result was likely caused by plasmablast contamination during the sorting process, as these transcripts were clonal in nature and were rapidly lost during the culture process. Interestingly despite inducing B cell activation and differentiation, the culture conditions did not induce CSR at either the genomic or protein level. Although a possibly unexpected result, since CD40 engagement ^53^ and IL-21 induce activation induced cytidine deaminase (AID) expression ^29^, which plays a key role in both the CSR and SHM pathways ^54^, this result does mirror a previously published result, which showed that IL-21 induced AID expression but no detectable SHM events ^29^. One possible explanation of our results comes from a recent publication that showed that CSR takes place much more readily in a hypoxic environment, likely recapitulating the environment in the germinal centre ^55^. Although this work was carried out using a murine model, it is possible that the same “checkpoint” exists in humans and provides a probable explanation for why we do not see CSR in our cultures. It would therefore be interesting to set up memory B cell cultures in such a hypoxic environment and test if we see CSR events and SHM taking place more readily than in our normoxic culture conditions. Importantly analysis of the Ig VH region highlighted that the culture conditions did not appear to induce SHM with levels of VH SHM staying relatively constant for each subset across the 10-day culture period. However, to fully confirm if the culture conditions impact the Ig locus monoclonal cell lines would need be cultured and then IgH sequencing carried out as was done by Kwakkenbos *et al.* ^24^.

Recently, the importance of IgM ^+^ memory B cells in mediating long term immunity in murine models has started to become apparent ^16, 17, 56^ and this work is now starting to be translated into humans. For example, recent work has shown that in humans the majority of circulating long lived tetanus toxoid specific memory B cells are IgM ^+^^57^. However, these cells are extremely rare and the authors did not delineate whether these cells were IgM ^+^ IgD ^+^ and/or IgM ^+^ IgD ^-^. By inducing robust expansion and differentiation of IgG ^+^, IgA ^+^, IgM ^+^ IgD ^+^ and IgM ^+^ IgD ^-^ cells, whilst not inducing CSR, the methods described in this manuscript will allow the responsiveness of rare memory B cells to be assessed irrespective of isotype. This could prove vitally important for the study of rare subsets including IgM expressing subsets such as B-1 cells.

In conclusion, our data show that a combination of IL-21, R848 and CD40 stimulation is optimal for the induction of purified memory B cell proliferation and differentiation into ASCs from a number of different memory B cell subsets. Importantly, characterisation of these culture conditions shows that they do not induce any undesired genotypic changes and are not biased towards any one memory B cell subset studied. Therefore, these conditions provide a valuable starting point for the investigation of memory B cell subset responses where proliferation is required to increase rare cell number, and differentiation is required to allow for functionality assessments.

## Data availability

All raw ELISA and FACS data, and the custom MATLAB script for DoE are available on OSF: http://doi.org/10.17605/OSF.IO/W96YG ^58^.

For sequencing data, accession numbers can be found in Table 4. It should be noted that access to samples must be requested from the Data Access Committee (DAC), whose contact details can be found on the EGA study page, accessible through the study accession number ( EGAS00001002633) or by emailing datasharing@sanger.ac.uk. The requester will be required to sign a data access agreement, which is in place to protect the identity of the sample donor via a managed access system.

**Table 4.** B cell sequencing accession numbers. EGA accession numbers and sample identifiers. EGA study accession number for all samples: EGAS00001002633.

Donor	Population	Expansion day	EGA sample accession number
1	IgM ^+^ IgD ^+^	0	EGAN00001588556
1	IgM ^+^ IgD ^-^	0	EGAN00001588558
1	IgM ^-^ IgD ^-^	0	EGAN00001588559
1	IgM ^+^ IgD ^+^	5	EGAN00001588560
1	IgM ^+^ IgD ^-^	5	EGAN00001588561
1	IgM ^-^ IgD ^-^	5	EGAN00001588562
1	IgM ^+^ IgD ^+^	10	EGAN00001588563
1	IgM ^+^ IgD ^-^	10	EGAN00001588564
1	IgM ^-^ IgD ^-^	10	EGAN00001588565
2	IgM ^+^ IgD ^+^	0	EGAN00001588549
2	IgM ^+^ IgD ^-^	0	EGAN00001588541
2	IgM ^-^ IgD ^-^	0	EGAN00001588542; EGAN00001588543
2	IgM ^+^ IgD ^+^	5	EGAN00001588575
2	IgM ^+^ IgD ^-^	5	EGAN00001588550
2	IgM ^-^ IgD ^-^	5	EGAN00001588551
2	IgM ^+^ IgD ^+^	10	EGAN00001588552
2	IgM ^+^ IgD ^-^	10	EGAN00001588554
2	IgM ^-^ IgD ^-^	10	EGAN00001588555

## Supplementary material

**Supplementary Figure 1: Gating strategy used for flow cytometry experiments including cell sorting, expansion phenotyping and MACS CD27 ^+^ selection purity analysis.** ( **A**) A time gate was used to make sure the sample was acquired without issue, ( **B**) lymphocytes were then gated on using a FSC-A vs SSC-A gate and ( **C**) single cells by setting FSC-H vs FSC-A gate. Memory B cells were then identified by gating on ( **D**) CD19 ^+^ live cells, which were negative for CD3, CD4 & CD14, followed by selection of ( **E**) CD27 ^+^ cells. Memory B cells and antibody secreting cells could then be phenotyped by looking at ( **F**) cell trace violet levels, ( **G**) IgD/IgM subsets, ( **H**) IgG expression and ( **I**) CD38 expression. ( **F**) Cell trace violet peaks were identified using the proliferation tool in Flow Jo version 9.

**Supplementary Figure 2: A wide screen of memory B cell differentiation stimuli identifies CD40 stimulation in combination with either IL-21 & CpG or IL-21 & R848 as the optimal stimulants.** 2×10 ^3^ memory B cells were cultured at a ratio of 4:1 with HV13280 cells and a checkerboard of cytokines and TLR agonists suggested to play a role in memory B cell expansion/differentiation at different concentrations. Heat map showing total Ig (IgG + IgA) secreted in culture supernatant as determined by ELISA after 5 days of culture. Data is representative of one donor.

**Supplementary Figure 3: A Design of Experiments approach identifies the effects of IL-21, R848, CpG ODN _2006_ & CD40 stimulation on IgG, IgM and IgA secretion by differentiating memory B cells.** 2×10 ^3^ memory B cells were cultured with all possible combinations of HV13280 cells, IL-21, R848 and CpG at three set concentrations for 10 days, after which total Ig (IgG, IgA & IgM) was measured in culture supernatant by ELISA. Each data point shows the average Ig output measured from 81 data points where the stimulant of interest was at a set concentration, but all other stimulants were being utilised in every possible combination (3 ^3^ for 3 donors = 81). "Errors bars" show the variation of Ig output measured across the 81 data points including the statistical error and, more importantly, the true effects of varying all other stimulant concentrations within their ranges. Thus these bars show how much the Ig output changes in response to three of the stimulants when the fourth one is fixed. Data shows a summary of three independent donors.

**Supplementary Figure 4: A Design of Experiments approach identifies a number of non-significant second order interaction effects on IgM, IgG and IgA secretion by differentiated memory B cells.** Following stimulation of 2×10 ^3^ memory B cells with iterative combinations of HV13280 cells, IL-21, CpG ODN _2006_ and R848 at three set concentrations over a 10-day culture period, IgG, IgM & IgA (ng/ml) secreted into the culture supernatant was measured by ELISA. ( **A**– **M**) A Matlab script was then used to determine the second order interaction effects of the chosen stimulants at each level on IgM, IgG & IgA secretion, these colour plots show the non-significant second order interactions. Data shows a summary of three independent donors.

**Supplementary Figure 5: The optimal Design of Experiments (DoE) expansion conditions induce high levels of Ig secretion.** 2×10 ^3^ memory B cells were cultured with combinations of ( **B**) HV13280 cells, IL-2 & R848, HV13280 cells, IL-21 & CpG as a comparator to the original screening results and the top DoE conditions identified, over 10 days. ( **A**) Total Ig (IgG, IgM & IgA) in culture supernatant was then measured by ELISA, errors bars show standard deviation. ( **C**) Statistical analysis of ELISA results (total Ig) as determined by two-way ANOVA with Tukey’s multiple comparison test. *p>0.05* (green), *p* ≤ 0.05 (yellow), ** *p* ≤ 0.01 (orange), *** *p* ≤ 0.001 (light red), and **** *p* ≤ 0.0001 (red). Data shows a summary of three independent donors.

**Supplementary Figure 6: Repeat Design of Experiments expansion/differentiation conditions do not induce undesirable Ig genotypic changes.** IgM ^+^ IgD ^+^, IgM ^+^ IgD ^-^, IgM ^-^ IgD ^-^ memory B cells were isolated by cell sorting and cultured using the optimised conditions. Samples were taken at baseline, day 5 and day 10 where Ig transcripts were analysed. ( **A**– **C**) Ig constant region transcripts measured from each subset at baseline, day 5 and day 10 of culture expressed as a percentage of isotype-specific Ig reads from the total Ig repertoire. ( **D**) VH SHM of each memory B cell subset across the three timepoints as a percentage of the total VH segment length at nucleotide level. ( **E**– **G**) Density plots of CDRH3 length for the individual subsets across the three time points, CDRH3 length is shown as nucleotides on the x axis.

## References

[1] LeBien TW Tedder TF: B lymphocytes: how they develop and function. *Blood.* 2008;112(5):1570–80. 10.1182/blood-2008-02-078071 18725575 PMC2518873

[2] De Silva NS Klein U: Dynamics of B cells in germinal centres. *Nat Rev Immunol.* 2015;15(3):137–48. 10.1038/nri3804 25656706 PMC4399774

[3] Crotty S: A brief history of T cell help to B cells. *Nat Rev Immunol.* 2015;15(3):185–9. 10.1038/nri3803 25677493 PMC4414089

[4] Victora GD Mesin L: Clonal and cellular dynamics in germinal centers. *Curr Opin Immunol.* 2014;28:90–6. 10.1016/j.coi.2014.02.010 24681449 PMC4037377

[5] Jelinek DF Splawski JB Lipsky PE: Human peripheral blood B lymphocyte subpopulations: functional and phenotypic analysis of surface IgD positive and negative subsets. *J Immunol.* 1986;136(1):83–92. 3484392

[6] Pascual V Liu YJ Magalski A: Analysis of somatic mutation in five B cell subsets of human tonsil. *J Exp Med.* 1994;180(1):329–39. 10.1084/jem.180.1.329 8006591 PMC2191579

[7] Tangye SG Liu YJ Aversa G: Identification of functional human splenic memory B cells by expression of CD148 and CD27. *J Exp Med.* 1998;188(9):1691–703. 10.1084/jem.188.9.1691 9802981 PMC2212517

[8] Klein U Rajewsky K Küppers R: Human immunoglobulin (Ig)M ^+^IgD ^+^ peripheral blood B cells expressing the CD27 cell surface antigen carry somatically mutated variable region genes: CD27 as a general marker for somatically mutated (memory) B cells. *J Exp Med.* 1998;188(9):1679–89. 10.1084/jem.188.9.1679 9802980 PMC2212515

[9] Tangye SG Good KL: Human IgM ^+^CD27 ^+^ B cells: memory B cells or "memory" B cells? *J Immunol.* 2007;179(1):13–9. 10.4049/jimmunol.179.1.13 17579014

[10] Klein U Küppers R Rajewsky K: Evidence for a large compartment of IgM-expressing memory B cells in humans. *Blood.* 1997;89(4):1288–98. 9028952

[11] Seifert M Przekopowitz M Taudien S: Functional capacities of human IgM memory B cells in early inflammatory responses and secondary germinal center reactions. *Proc Natl Acad Sci U S A.* 2015;112(6):E546–55. 10.1073/pnas.1416276112 25624468 PMC4330750

[12] Buisman AM de Rond CG Oztürk K: Long-term presence of memory B-cells specific for different vaccine components. *Vaccine.* 2009;28(1):179–86. 10.1016/j.vaccine.2009.09.102 19799844

[13] Amanna IJ Carlson NE Slifka MK: Duration of humoral immunity to common viral and vaccine antigens. *N Engl J Med.* 2007;357(19):1903–15. 10.1056/NEJMoa066092 17989383

[14] Pauli NT Henry Dunand CJ Wilson PC: Exploiting human memory B cell heterogeneity for improved vaccine efficacy. *Front Immunol.* 2011;2:77. 10.3389/fimmu.2011.00077 22566866 PMC3342318

[15] Mroczek ES Ippolito GC Rogosch T: Differences in the composition of the human antibody repertoire by B cell subsets in the blood. *Front Immunol.* 2014;5:96. 10.3389/fimmu.2014.00096 24678310 PMC3958703

[16] Krishnamurty AT Thouvenel CD Portugal S: Somatically Hypermutated *Plasmodium*-Specific IgM ^+^ Memory B Cells Are Rapid, Plastic, Early Responders upon Malaria Rechallenge. *Immunity.* 2016;45(2):402–14. 10.1016/j.immuni.2016.06.014 27473412 PMC5118370

[17] Narváez CF Feng N Vásquez C: Human rotavirus-specific IgM Memory B cells have differential cloning efficiencies and switch capacities and play a role in antiviral immunity *in vivo*. *J Virol.* 2012;86(19):10829–40. 10.1128/JVI.01466-12 22855480 PMC3457288

[18] Meffre E Louie A Bannock J: Maturational characteristics of HIV-specific antibodies in viremic individuals. *JCI Insight.* 2016;1(3): pii: e84610. 10.1172/jci.insight.84610 27152362 PMC4854302

[19] Sok D van Gils MJ Pauthner M: Recombinant HIV envelope trimer selects for quaternary-dependent antibodies targeting the trimer apex. *Proc Natl Acad Sci U S A.* 2014;111(49):17624–9. 10.1073/pnas.1415789111 25422458 PMC4267403

[20] Bardelli M Alleri L Angiolini F: *Ex vivo* analysis of human memory B lymphocytes specific for A and B influenza hemagglutinin by polychromatic flow-cytometry. *PLoS One.* 2013;8(8):e70620. 10.1371/journal.pone.0070620 23976947 PMC3744578

[21] Moody MA Haynes BF: Antigen-specific B cell detection reagents: use and quality control. *Cytometry A.* 2008;73(11):1086–92. 10.1002/cyto.a.20599 18613115 PMC3105373

[22] Heath E Begue-Pastor N Chaganti S: Epstein-Barr virus infection of naïve B cells *in vitro* frequently selects clones with mutated immunoglobulin genotypes: implications for virus biology. *PLoS Pathog.* 2012;8(5):e1002697. 10.1371/journal.ppat.1002697 22589726 PMC3349760

[23] Dorner M Zucol F Berger C: Distinct *ex vivo* susceptibility of B-cell subsets to epstein-barr virus infection according to differentiation status and tissue origin. *J Virol.* 2008;82(9):4400–12. 10.1128/JVI.02630-07 18321980 PMC2293034

[24] Kwakkenbos MJ Diehl SA Yasuda E: Generation of stable monoclonal antibody-producing B cell receptor-positive human memory B cells by genetic programming. *Nat Med.* 2010;16(1):123–8. 10.1038/nm.2071 20023635 PMC2861345

[25] Kwakkenbos MJ van Helden PM Beaumont T: Stable long-term cultures of self-renewing B cells and their applications. *Immunol Rev.* 2016;270(1):65–77. 10.1111/imr.12395 26864105 PMC4755196

[26] Huang J Kang BH Ishida E: Identification of a CD4-Binding-Site Antibody to HIV that Evolved Near-Pan Neutralization Breadth. *Immunity.* 2016;45(5):1108–21. 10.1016/j.immuni.2016.10.027 27851912 PMC5770152

[27] Henn AD Rebhahn J Brown MA: Modulation of single-cell IgG secretion frequency and rates in human memory B cells by CpG DNA, CD40L, IL-21, and cell division. *J Immunol.* 2009;183(5):3177–87. 10.4049/jimmunol.0804233 19675172 PMC2765874

[28] Cao Y Gordic M Kobold S: An optimized assay for the enumeration of antigen-specific memory B cells in different compartments of the human body. *J Immunol Methods.* 2010;358(1–2):56–65. 10.1016/j.jim.2010.03.009 20302874

[29] Ettinger R Sims GP Fairhurst AM: IL-21 induces differentiation of human naive and memory B cells into antibody-secreting plasma cells. *J Immunol.* 2005;175(12):7867–79. 10.4049/jimmunol.175.12.7867 16339522

[30] Pinna D Corti D Jarrossay D: Clonal dissection of the human memory B-cell repertoire following infection and vaccination. *Eur J Immunol.* 2009;39(5):1260–70. 10.1002/eji.200839129 19404981 PMC3864550

[31] Karahan GE Eikmans M Anholts JD: Polyclonal B cell activation for accurate analysis of pre-existing antigen-specific memory B cells. *Clin Exp Immunol.* 2014;177(1):333–40. 10.1111/cei.12305 24611883 PMC4089183

[32] Jourdan M Caraux A De Vos J: An *in vitro* model of differentiation of memory B cells into plasmablasts and plasma cells including detailed phenotypic and molecular characterization. *Blood.* 2009;114(25):5173–81. 10.1182/blood-2009-07-235960 19846886 PMC2834398

[33] Geffroy-Luseau A Chiron D Descamps G: TLR9 ligand induces the generation of CD20+ plasmablasts and plasma cells from CD27+ memory B-cells. *Front Immunol.* 2011;2:83. 10.3389/fimmu.2011.00083 22566872 PMC3342082

[34] Crotty S Aubert RD Glidewell J: Tracking human antigen-specific memory B cells: a sensitive and generalized ELISPOT system. *J Immunol Methods.* 2004;286(1–2):111–22. 10.1016/j.jim.2003.12.015 15087226

[35] Néron S Racine C Roy A: Differential responses of human B-lymphocyte subpopulations to graded levels of CD40-CD154 interaction. *Immunology.* 2005;116(4):454–63. 10.1111/j.1365-2567.2005.02244.x 16313359 PMC1802436

[36] Berkowska MA Driessen GJ Bikos V: Human memory B cells originate from three distinct germinal center-dependent and -independent maturation pathways. *Blood.* 2011;118(8):2150–8. 10.1182/blood-2011-04-345579 21690558 PMC3342861

[37] Jahnmatz M Kesa G Netterlid E: Optimization of a human IgG B-cell ELISpot assay for the analysis of vaccine-induced B-cell responses. *J Immunol Methods.* 2013;391(1–2):50–9. 10.1016/j.jim.2013.02.009 23454005

[38] Weiss GE Ndungu FM McKittrick N: High efficiency human memory B cell assay and its application to studying *Plasmodium falciparum*-specific memory B cells in natural infections. *J Immunol Methods.* 2012;375(1–2):68–74. 10.1016/j.jim.2011.09.006 21963949 PMC3253904

[39] Bernasconi NL Traggiai E Lanzsavecchia A: Maintenance of serological memory by polyclonal activation of human memory B cells. *Science.* 2002;298(5601):2199–202. 10.1126/science.1076071 12481138

[40] Bernasconi NL Onai N Lanzavecchia A: A role for Toll-like receptors in acquired immunity: up-regulation of TLR9 by BCR triggering in naive B cells and constitutive expression in memory B cells. *Blood.* 2003;101(11):4500–4. 10.1182/blood-2002-11-3569 12560217

[41] Scholzen A Nahrendorf W Langhorne J: Expansion of IgG+ B-cells during mitogen stimulation for memory B-cell ELISpot analysis is influenced by size and composition of the B-cell pool. *PLoS One.* 2014;9(7):e102885. 10.1371/journal.pone.0102885 25050555 PMC4106867

[42] Walsh PN Friedrich DP Williams JA: Optimization and qualification of a memory B-cell ELISpot for the detection of vaccine-induced memory responses in HIV vaccine trials. *J Immunol Methods.* 2013;394(1–2):84–93. 10.1016/j.jim.2013.05.007 23707324 PMC3736720

[43] Watson SJ Welkers MR Depledge DP: Viral population analysis and minority-variant detection using short read next-generation sequencing. *Philos Trans R Soc Lond B Biol Sci.* 2013;368(1614):20120205. 10.1098/rstb.2012.0205 23382427 PMC3678329

[44] Lefranc MP Giudicelli V Ginestoux C: IMGT, the international ImMunoGeneTics information system. *Nucleic Acids Res.* 2009;37(Database issue):D1006–12. 10.1093/nar/gkn838 18978023 PMC2686541

[45] Altschul SF Gish W Miller W: Basic local alignment search tool. *J Mol Biol.* 1990;215(3):403–10. 10.1016/S0022-2836(05)80360-2 2231712

[46] Wei C Jung J Sanz I: OMIP-003: phenotypic analysis of human memory B cells. *Cytometry A.* 2011;79(11):894–6. 10.1002/cyto.a.21112 21796774 PMC3199331

[47] Kaminski DA Wei C Qian Y: Advances in human B cell phenotypic profiling. *Front Immunol.* 2012;3:302. 10.3389/fimmu.2012.00302 23087687 PMC3467643

[48] Horns F Vollmers C Croote D: Lineage tracing of human B cells reveals the *in vivo* landscape of human antibody class switching. *eLife.* 2016;5: pii: e16578. 10.7554/eLife.16578 27481325 PMC4970870

[49] Linterman MA Beaton L Yu D: IL-21 acts directly on B cells to regulate Bcl-6 expression and germinal center responses. *J Exp Med.* 2010;207(2):353–63. 10.1084/jem.20091738 20142429 PMC2822609

[50] Kuraoka M Schmidt AG Nojima T: Complex Antigens Drive Permissive Clonal Selection in Germinal Centers. *Immunity.* 2016;44(3):542–52. 10.1016/j.immuni.2016.02.010 26948373 PMC4794380

[51] Shaw R Festing MF Peers I: Use of factorial designs to optimize animal experiments and reduce animal use. *ILAR J.* 2002;43(4):223–32. 10.1093/ilar.43.4.223 12391398

[52] O'Neill LA Golenbock D Bowie AG: The history of Toll-like receptors - redefining innate immunity. *Nat Rev Immunol.* 2013;13(6):453–60. 10.1038/nri3446 23681101

[53] Muramatsu M Sankaranand VS Anant S: Specific expression of activation-induced cytidine deaminase (AID), a novel member of the RNA-editing deaminase family in germinal center B cells. *J Biol Chem.* 1999;274(26):18470–6. 10.1074/jbc.274.26.18470 10373455

[54] Stavnezer J Guikema JE Schrader CE: Mechanism and regulation of class switch recombination. *Annu Rev Immunol.* 2008;26:261–92. 10.1146/annurev.immunol.26.021607.090248 18370922 PMC2707252

[55] Abbott RK Thayer M Labuda J: Germinal Center Hypoxia Potentiates Immunoglobulin Class Switch Recombination. *J Immunol.* 2016;197(10):4014–20. 10.4049/jimmunol.1601401 27798169 PMC5123804

[56] Yates JL Racine R McBride KM: T cell-dependent IgM memory B cells generated during bacterial infection are required for IgG responses to antigen challenge. *J Immunol.* 2013;191(3):1240–9. 10.4049/jimmunol.1300062 23804710 PMC3720767

[57] Della Valle L Dohmen SE Verhagen OJ: The majority of human memory B cells recognizing RhD and tetanus resides in IgM ^+^ B cells. *J Immunol.* 2014;193(3):1071–9. 10.4049/jimmunol.1400706 24965774 PMC4105240

[58] Muir L: Optimisation of *ex vivo* memory B Cell expansion/differentiation for interrogation of rare peripheral memory B cell subset responses Raw Data.2017 Data Source 10.12688/wellcomeopenres.11386.2 PMC5843844 29588920

