# Supplementary material for: Optimisation of ex vivo memory B cell expansion/differentiation for interrogation of rare peripheral memory B cell subset responses
Source: Wellcome Open Res. 2018 Jan 24;2:97. Originally published 2017 Oct 5. [Version 2] doi: 10.12688/wellcomeopenres.11386.2 (PMC5843844; doi:10.12688/wellcomeopenres.11386.2)
Supplement: Peer review history

# Peer review: Referee response for version 2

**DOI:** 10.21956/wellcomeopenres.14259.r30180

**Published:** 2018-03-08

**Authors:** Elise Landais

**Affiliations:** 1 The International AIDS Vaccine Initiative, Neutralizing Antibody Center, La Jolla, CA, USA; 2 Department of Immunology and Microbiology, The Scripps Research Institute, La Jolla, CA, USA

The Authors have adequately addressed all reviewers comments, adding requested data and supporting information, editing figures and text accordingly.

 I would like to thanks the editors for giving me the opportunity to review and the authors for thoroughly addressing the reviewers comments. This is an interesting paper that will undoubtedly help the broader scientific community advance the analyses of memory B cell populations.

I have read this submission. I believe that I have an appropriate level of expertise to confirm that it is of an acceptable scientific standard.

# Peer review: Referee response for version 2

**DOI:** 10.21956/wellcomeopenres.14259.r30181

**Published:** 2018-02-15

**Authors:** Adrian B. McDermott, David J. Leggat

**Affiliations:** 1 Vaccine Immunology Program, Vaccine Research Center, National Institute of Allergy and Infectious Diseases, Bethesda, MD, USA

The authors of the manuscript "Optimisation of *ex vivo *memory B cell expansion/differentiation for interrogation of rare peripheral memory B cell subset responses" have satisfactorily addressed each of the points outlined in the critique from October 31st 2017.  

 We would like to thanks the editors and authors for giving us the opportunity to review and also addressing our points sequentially and eloquently.  This is an interesting paper that will further advance the analyses of memory B cell populations and in the design of critical end point clinical trial analyses.

We have read this submission. We believe that we have an appropriate level of expertise to confirm that it is of an acceptable scientific standard.

# Peer review: Referee response for version 1

**DOI:** 10.21956/wellcomeopenres.12291.r26745

**Published:** 2017-10-31

**Authors:** Adrian B. McDermott, David J. Leggat

**Affiliations:** 1 Vaccine Immunology Program, Vaccine Research Center, National Institute of Allergy and Infectious Diseases, Bethesda, MD, USA

In this manuscript “Optimisation of *ex vivo *memory B cell expansion/differentiation for interrogation of rare peripheral memory B cell subset responses” by Muir *et al*, the group tackles the methodologies of how to interrogate low frequency memory B cell responses following vaccination. Reminiscent of the trajectory that T cell immunobiology embarked upon some 25 years ago, the investigation of *ex-vivo* B cells (using tetrameric complexes to specifically sort phenotypically characterized antigen specific B cells) is preferable to stimulation and culturing techniques that have the potential to alter the target B cell populations. Here Muir *et al *test a matrix of stimulation techniques that would potentially minimally affect the target memory cell populations while amplifying the frequency to enable appropriate investigation. 

 Overall this is an interesting approach that, in the main, is well articulated and presented with relevant experiments. The methodology would be of use to the general vaccine community particularly when specific proteins are unavailable or memory B cells are in low frequency. Below are some suggestions and questions for the authors to consider.  

 The authors lay out their rationale and approach in the Introduction adequately but may include some more details: investigation of subsets of human B-1 cells, which would strengthen the authors point of studying rare IgM B cell subtypes. Also, the presence of B cell subtypes that are not easily identified by fluorophore conjugated proteins (probes) using flow cytometry such as those that are poly-reactive or problems in fluorescently labeling polysaccharides. The use of retroviral transduction (Kwakkenbos *et al*, Nat Med 2010) should also be accounted for when mentioning EBV immortalization. An alternative reference to Moir *et al* (ref 21) should be used to make this point regarding the use of conjugated protein probes for the identification of B cell populations, as this misrepresents this particular paper. In the 5 ^th^ paragraph of the Introduction the authors correctly cite the use of culture methods in the identification of HIV neutralizing antibodies and the need to perform B cell ELISPOT in the occasion that antigen specific proteins are unavailable. However, the statement *“there has been no study aimed at identifying memory B cell expansion/differentiation across different subsets”* should be deleted and the inclusion of indicated references (Jahnmatz, M. *et al*.  Journal of immunological methods **391,** 50–9 (2013) and Berkowska, M. *et al.*  Blood **118,** 2150–8 (2011)) should be cited and included, then comment upon why this manuscript is novel in the context of the previously published memory B cell analyses. 

 When laying out the methods being employed it may have been useful to comment upon why certain stimuli were omitted, as well as included e.g. Poke weed mitogen (PWM). Furthermore, perhaps including in Table 1, the targets of each stimuli, such as the TLR interactions, would be informative to the readers as they select the stimuli most applicable for use. The preferential amplification/proliferation of certain B cell subsets would have added to the novelty of the manuscript and this point was noted in the Introduction. However, the omission of CD21 from the flow cytometry staining and sorting panel resulted in a lost opportunity to monitor the relative frequencies of “resting” versus “activated” at baseline in combination with CD27 (ref 21, Moir *et al*). It has been discussed elsewhere that *in-vitro* stimulation protocols favour the expansion of the “resting” memory B cell populations, which has implications in vaccine immune-monitoring. 

 The results are well laid out and communicated sequentially to the benefit of the reader. Minor points: the authors mention CD154 without explaining that this is the ligand for CD40, which is important to the conclusions of the manuscript. Perhaps the inclusion of a gating strategy as a supplemental on baseline samples rather than older co-cultures would be conventional to demonstrate the specificity of the flow cytometry panel. Results paragraph 6: “As well as having significant first order effects, the combination of IL-21 and CD40…” Maybe the authors would like to include “CD40 stimulation” to be consistent? In Figure 4, Day 10 F, can the authors comment upon the reappearance of IgM?  Figure 5: Another supplemental figure that includes the primers used for the determination of the SHM or reference in the text. If these are the same primers as Tiller *et al J. Immunol. Methods* **329,** 112–24 (2008) then indicate. Panels D and E are labeled the same IgM+IgD+, presumably E should be IgM+IgD-? Can the authors explain in the Results or Discussion why they used general CDRH3 length and not a specific vaccine epitope repertoire response to illustrate the accumulation of point mutations within the CDRH2 and 3 locus? This is important for the overall message of the manuscript and not to determine experimentally the accumulation of random point mutations would be a critical piece of data, should the method be deployed to analyze the evolution of the memory B cell repertoire following vaccination -  especially critical for the analyses of the eOD-GT8 IgM responses cited in the Discussion. This should be reanlaysed, corrected or commented upon in the discussion. Overall the discussion should be shortened and more focused upon the positive impact and applications of the method described.

We have read this submission. We believe that we have an appropriate level of expertise to confirm that it is of an acceptable scientific standard, however we have significant reservations, as outlined above.

## References

[1] JahnmatzMKesaGNetterlidEBuismanAMThorstenssonRAhlborgN: Optimization of a human IgG B-cell ELISpot assay for the analysis of vaccine-induced B-cell responses. *J Immunol Methods*.2013;391(1-2) : 10.1016/j.jim.2013.02.009 50-9 10.1016/j.jim.2013.02.009 23454005

[2] BerkowskaMADriessenGJBikosVGrosserichter-WagenerCStamatopoulosKCeruttiAHeBBiermannKLangeJFvan der BurgMvan DongenJJvan ZelmMC: Human memory B cells originate from three distinct germinal center-dependent and -independent maturation pathways. *Blood*.2011;118(8) : 10.1182/blood-2011-04-345579 2150-8 10.1182/blood-2011-04-345579 21690558PMC3342861

[3] GriffinDOHolodickNERothsteinTL: Human B1 cells in umbilical cord and adult peripheral blood express the novel phenotype CD20+ CD27+ CD43+ CD70-. *J Exp Med*.2011;208(1) : 10.1084/jem.20101499 67-80 10.1084/jem.20101499 21220451PMC3023138

[4] LeggatDJKhaskhelyNMIyerASMosakowskiJThompsonRSWeinandyJDWesterinkMA: Pneumococcal polysaccharide vaccination induces polysaccharide-specific B cells in adult peripheral blood expressing CD19⁺CD20⁺CD3⁻CD70⁻CD27⁺IgM⁺CD43⁺CD5⁺/⁻. *Vaccine*.2013;31(41) : 10.1016/j.vaccine.2013.07.030 4632-40 10.1016/j.vaccine.2013.07.030 23911852PMC3810315

[5] KwakkenbosMJDiehlSAYasudaEBakkerAQvan GeelenCMLukensMVvan BleekGMWidjojoatmodjoMNBogersWMMeiHRadbruchAScheerenFASpitsHBeaumontT: Generation of stable monoclonal antibody-producing B cell receptor-positive human memory B cells by genetic programming. *Nat Med*.2010;16(1) : 10.1038/nm.2071 123-8 10.1038/nm.2071 20023635PMC2861345

# Response

**Published:** 2018-01-18

**Authors:** Luke Muir

**Affiliations:** Imperial College London, UK

In response to the reviewers' comments the Introduction has been edited to discuss potential applications of the work for identification of poly-reactive IgM memory B cells and potential for use with the B-1 B cell subset. Similarly a brief section discussing the Bcl-6 and Bcl-xL retroviral transduction system for long term memory B cell cultures has been added to the introduction.

We have also updated the Moir reference with regards to flow sorting as this was a miss reference that was not picked up on proof reading and has been removed, we thank the reviewers for picking up on this. The Berkowska *et al* and Jahnmatz *et al* references have been cited and the text edited to make it clear that the novelty of the manuscript lies in the identification of memory B cell differentiation conditions for the IgM ^+^ IgD ^+^, IgM ^+^ IgD ^-^ and IgM ^-^ IgD ^-^ subsets. The Berkowska *et al* publication shows a detailed analysis of the potential origins of different memory B cell subsets but does not look at the use of the *ex vivo* memory B cell culture technique for these subsets. In contrast the Jahnmatz *et al *publication focuses on the optimisation of memory B cell culture conditions but focuses solely on IgG ^+^ memory B cells. The potential applications and importance of defining these conditions are also discussed.

We think that the targets of each of the stimuli would be a welcome addition to Table 1 and have added these. The flow cytometry panel was a basic memory B cell panel selected to mirror the magnetic memory B cell isolation kit. In the analysis of future vaccine studies we agree that it would be worthwhile to include CD21 in the panel. We are also looking at including CD80, PD-L2 and CD73 which have been used in murine models to identify the differentiation capabilities of memory B cell subsets and could have a similar role in humans (Anderson *et al*, JEM, 2007. Tomayko *et al*, JI, 2010). The manuscript text has been edited to ensure that “CD40 stimulation” is used consistently. CD154 is listed as CD40-ligand in both the introduction and the discussion.

With regards to the reappearance of the IgM ^+^ IgD ^-^ memory B cell population at day 10, we would first like to point out that this population is reduced considerably when compared to the baseline sample. However, the reviewers are correct in pointing out that although the population is absent at day 5 of culture there appears to be a clearly defined IgM ^+^ IgD ^-^ population at day 10 of culture. One possible explanation for this discrepancy could be that the surface IgM levels are decreased as the cells undergo rapid proliferation, similar to the germinal centre reaction. Then at day 10 when the IL-21 and R848 concentrations may have become limiting the cells are less proliferative and start to re express surface IgM. This could also explain a similar slight increase in the IgG ^+^ population although this was not as clearly defined. This hypothesis could be tested by the re-addition of fresh IL-21 and R848 at later time points.

As mentioned to reviewer 1 the primer sequences have been added as a table to the methods section.

Figure 5E has been edited to read “IgM ^+^ IgD ^-^”.

Our original analysis which solely focused on CDRH3 length was clearly an oversight with the paper and we have now re analysed the data to look at total VH region SHM across the 10 day culture period. This data has been inserted into the paper and shows that the SHM levels do not appear to change for any of the 3 subsets across the 10 day culture period, with potential limitations and alternatives to this approach discussed in the discussion. As suggested the discussion has been reviewed for potential revisions to make it more concise and focus upon the benefits of this method.

# Peer review: Referee response for version 1

**DOI:** 10.21956/wellcomeopenres.12291.r26746

**Published:** 2017-10-16

**Authors:** Elise Landais

**Affiliations:** 1 The International AIDS Vaccine Initiative, Neutralizing Antibody Center, La Jolla, CA, USA; 2 Department of Immunology and Microbiology, The Scripps Research Institute, La Jolla, CA, USA

This manuscript “Optimization of ex vivo memory B-cell expansion/differentiation for interrogation of rare peripheral memory B-cell subset responses” describes a very thorough and systematic screening of various reagents combinations and concentrations with the goal to optimize culture conditions for the *ex-vivo* expansion and antibody secreting cells (ACS) differentiation of peripheral B-cell subsets. Reagents were selected based on literature review and culture conditions were optimized in a two-step process to first quickly identify the best combination and to then fine-tuned the concentrations and ratio required for optimal expansion and Ig secretion of each B-cell subset, in an extensive matrix format complemented by a comprehensive statistical analysis to evaluate the impact of each factor. Finally, they verified that the phenotype and genotype of different B-cell subsets were not altered during the optimized *ex-vivo* culture process.

 The manuscript is well written, the study rational is sound, the experimental strategy is logical and well-explained, and the results are presented in a clear manner with main figures displaying all key relevant results and additional information made available in supplementary material. To a large extend the conclusions are adequate in regards of the presented data, with reserved regarding Figure 4M-N conclusions and the last paragraph of the results section (see Major Points).

 Overall, this study should be of great interest to B cell immunologists interested in characterizing human Ab responses to infection or in other disease models where sample size and availability is often limiting.

 Issues that should be addressed:

 Major Point# 1- In the paragraph of the results section, the authors describes how they assessed whether their optimized culture conditions induced somatic hypermutations (SHM) which would alter the genotype and functional characteristics of the secreted Igs. They chose to evaluate CDRH3 length variation as an indicator for SHM and INDELs taking place. However, INDELs are often non-productive due to off-frame sequence or structurally unstable/aggregating Abs, such that variations in CDRH3 length are rather rare occurences compared to single nucleotides or amino-acid variations which are more frequently observed during the SHM process, not only in the CDR3 but also at other CDRs and SHM hot-spots as well.

 Furthermore, the Methods section seem to imply (although it is not very clear, also see Minor Point 1 below) that the NGS covered the entire V-region (VDJ) and only the CRD3 region. The authors should therefore provide additional SHM data regarding the V and J genes to truly support their conclusions.

 Major Point#2. In the third sub-headed section of the Results, the authors conclude from Figure 4M-N that “ *CD38 and Ig expression levels changed relative to the number of divisions that had taken place [during the ex-vivo-culture]…suggesting that as the cells proliferate, they progressively differentiate and lose Ig expression”.* However, the data presented Figure 4 M-N do not really support these statements as the level of Ig in particular, is fairly equivalent for each “peak” (= cell division). Maybe a blow up of the lower values would help seeing the significance of this statement.

 Minor Point #1 - The sequence of primers used for the B-cell next generation sequencing should be provided in a table to ensure full reproducibility of the experiments as well as comparison with other primers sets already published.

 Minor Point #2 – Figure 5D-G and Supplementary Figure 6D-F present CDR3 length data but the fact that these are Heavy Chain sequencing data is not stated clearly throughout the paper and in the figures. All mentions of CDR3 should be changed to CDRH3 or HCDR3.

 Minor Point #3- It is mentioned that 10 donors independent donors were used for this study and figures present either data for one representative donor or a summary of 2 to 3 donors. A small figure or text mention of the extent of the variability between donors would be appreciated.

 Note that I could not access the NGS data using the provided links, yet I suggest the above mentioned information regarding Ig NGS be included in the main manuscript.

I have read this submission. I believe that I have an appropriate level of expertise to confirm that it is of an acceptable scientific standard, however I have significant reservations, as outlined above.

# Response

**Published:** 2018-01-18

**Authors:** Luke Muir

**Affiliations:** Imperial College London, UK

**Response to Major Point 1:** The sequencing did cover the whole VDJ region and we have now completed analysis of the SHM levels withing the VH region. This data has been added to the manuscript and highlights that the levels of SHM do not appear to change over the 10-day culture period. 

**Response to Major Point 2:** Having reviewed the data the text been edited to reflect this point. The text now reads “Interestingly although surface Ig expression rapidly decreased, CD38 expression levels changed relative to the number of divisions that had taken place (Figures 4M and N). This suggests that as the cells proliferate they progressively differentiate towards ASCs.” We have also altered Figure 4 which hopefully makes the drop in surface Ig expression levels easier to interpret.

**Response to Minor Point 1:** We have added a table highlighting the sequences of the NGS primers to the methods section.

**Response to Minor Point 2: **This was clearly an oversight in the manuscript and the text has now been updated to ensure CDRH3 is used consistently.

**Response to Minor Point 3:** Due to the rarity of some subsets it was not possible to use the same 3 donors throughout the whole manuscript. As suggested a section that reads as follows has been added into the methods section: “Due to the rarity of some subsets the same donors could not be used throughout the whole study. Therefore memory B cells were isolated from 10 different donors and replicates from 1-3 donors used per individual experiment. This meant that inter donor variability was measured throughout each experiment but not between different experiments. However, it should be noted that all isolated memory B cells and subsets from all donors were well within the expected normal range”

With regards to the NGS data, the link for the study accession in the legend for table 3 takes you to a page where you can request access to all the sequence samples listed in table 3. For ethical purposes, it is not possible to publish the human clinical trial participant sequencing data openly on the internet but access can be requested.
